# A vascular anomaly of the iliac artery in a patient with VATER association

**DOI:** 10.1002/ccr3.1444

**Published:** 2018-02-27

**Authors:** Takaaki Nakano, Tomoya Asaka, Masaaki Takemoto, Tomonori Imamura, Toshitaka Ito

**Affiliations:** ^1^ Department of Emergency Medicine Shinyurigaoka General Hospital 255 Furusawatuko Asou‐ku Kawasaki Kanagawa 215‐0026 Japan; ^2^ Department of Emergency Medicine Yokohama Sakae Kyousai Hospital 132 Katsura Sakae‐ku Yokohama Kanagawa 247‐8581 Japan

**Keywords:** Gastrointestinal bleeding, iliac artery, VATER association

## Abstract

In patients with VATER association, some have vascular anomaly that makes procedure difficult. Pretreatment CT angiography should be necessary for the patients with VATER association's feature.

## Introduction

VATER association has been coined from a random combination of the initials of vertebral defects, anal atresia, tracheoesophageal fistula with esophageal atresia, and radial or renal dysplasia. At least two anomalies indicate a definitive diagnosis of VATER association.

In a patient who underwent interventional radiology for minor intestinal bleeding, we observed a traveling abnormality of the right iliac artery. This patient had the defective right external iliac artery and migration of internal iliac artery directly to the femoral artery. VATER association is referred to as VACTERL association when it is combined with heart malformations and limb anomalies. From the embryological viewpoint, the mesoderm is derived from the heart and blood vessels. Hence, the combination of this vascular malformation was considered one of the phenotypes of VATER association. A common iliac artery abnormality may be added as a new malformation complicated with VATER association.

The term “VATER” association was coined from the combination of the initials for vertebral defects (V), anal atresia (A), tracheoesophageal fistula with esophageal atresia (TE), and radial or renal dysplasia (R). The presence of at least two anomalies indicates a definitive diagnosis of VATER association, according to Quan and Smith (1972) 8. When VATER association occurs with heart malformations and limb anomalies, it is called VACTERL association 1. Only one case of an external iliac artery‐deficient vascular malformation occurring with VACTERL association has been previously reported 2. This report described an abnormality of the right external iliac artery in a patient with VATER association who needed renal transplantation, and showed the usefulness of reconstructed computed tomography (CT) for selecting the anastomotic vessel. From an embryological viewpoint, the heart and blood vessels are derived from the mesoderm. We observed a traveling abnormality of the right iliac artery in a patient who had VATER association and underwent interventional radiology for minor intestinal bleeding. Hence, this combination with a vascular malformation was considered one of the VACTERL association phenotypes.

## Case Presentation

**Figure 1:**
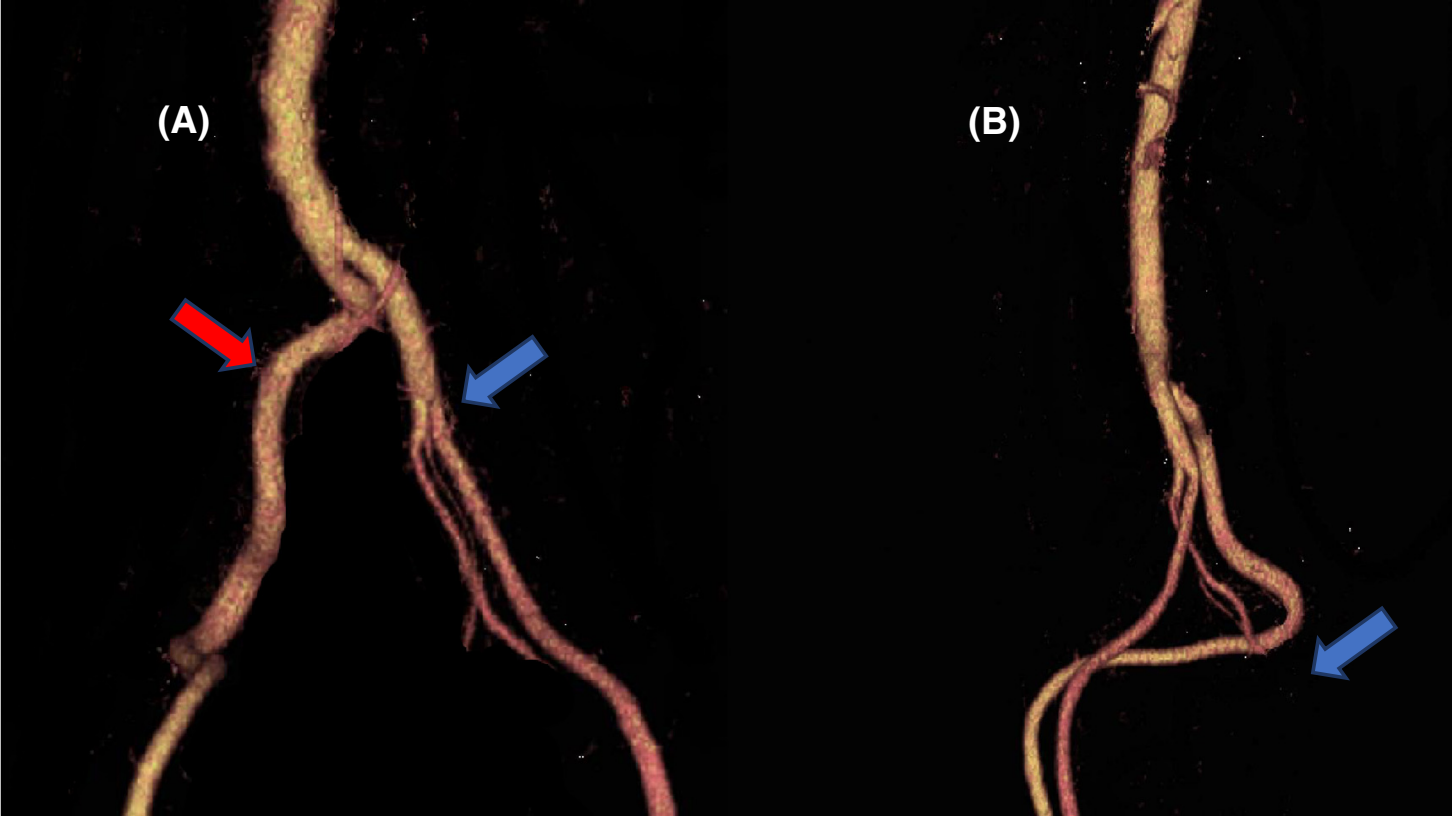
Three‐dimensional CT of aorta. (A) The abdominal aortic branch in front of the fourth lumbar vertebra. The left common iliac artery becomes a normal branch of the internal iliac artery and external iliac artery (blue arrow). However, the right common iliac artery is deficient in the external iliac artery (red arrow), as it flows directly to the internal iliac artery and descends along the front of the sacrum. (B) Lateral view of aorta. The angle of the abnormal blood vessel on the right is 34° (blue arrow).

A 40‐year‐old man presented with fresh hemorrhagic stool. Colonoscopy and upper gastrointestinal endoscopy were performed, but the bleeding site was not clear in the visible range of the stomach, duodenum, and colon. On hospital day 2, capsule endoscopy was performed, and bleeding from the small intestine was detected. On hospital day 3, the patient developed shock due to massive bleeding. Hence, emergency transcatheter arterial embolization was performed. He had a history of surgery for anal atresia and small bowel stoma as a neonate, and he required hemodialysis because of chronic renal failure with bilateral kidney malformations. Computed tomography performed before transcatheter arterial embolization showed that the anatomy of the left common iliac artery was normal. However, the right external iliac artery was defective. Blood from the right common iliac artery flowed directly into a traveling vessel: the internal iliac artery. The blood vessel descended at an acute angle to the pelvic floor, and then, it rose and flowed into the femoral artery (Fig. 1). We chose to insert the catheter from the left femoral artery and then further selectively advanced the catheter to the superior mesenteric artery, which is the responsible vessel for gastrointestinal bleeding. Regarding the superior mesenteric artery, the branch from the aorta was at the same level as the celiac artery, and the right hepatic artery originated from the superior mesenteric artery. But angiography did not show a clear extravasation from either branch of the superior mesenteric artery. Small intestinal endoscopy was performed on hospital day 8. However, it was impossible to advance the endoscope because the small intestinal adhesion was very strong. Thereafter, there was no massive bleeding. He was discharged home on hospital day 20 after red blood cell transfusion. Later, he was hospitalized again for small intestinal resection. In the abdominal cavity, the small intestine was firmly adhered. First, 30 cm of the adhered small intestine was resected. Then, small intestinal endoscopy was performed through the small bowel stump. There were two diverticula on the oral side, but no obvious bleeding was found. A submucosal cystic lesion was found during small intestinal resection, and it was judged to be a bleeding site due to redness.

**Figure 2:**
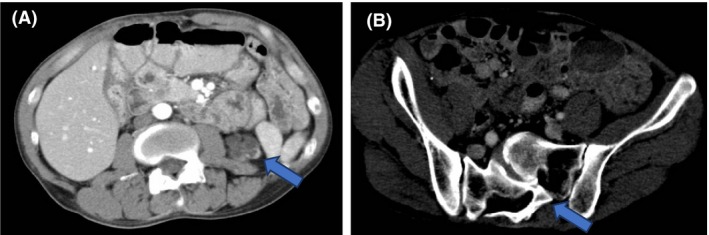
A CT image showing the characteristic of VATER association. (A) Renal malformations (hypoplasia of the left kidney: blue arrow, right kidney aplasia) and (B) vertebral body abnormality are recognized. These are two of the five signs that comprise VATER association.

Later, it was confirmed that he met the diagnostic criteria for VATER association because of the deformity of the sacrum, right aplastic kidney, left hypoplastic kidney (Fig. 2), and history of anal atresia.

## Discussion

Khoury et al. 1 proposed the concept of VACTERL association by adding cardiac malformations and limb anomalies to the five signs of VATER association. Further, VACTERL‐H4, which adds hydrocephalus to VACTERL association, was proposed in 1983 7. Regarding gene anomalies, mutations of the ZIC3 gene have been observed in VACTERL X; however, consistent gene mutations are unclear in VATER/VACTERL association. Conditions that can be combined with VATER association have been newly discovered, and there are few reports of the involvement of a running branch of the external iliac artery. Tay et al. 2 reported a patient with right external iliac artery hypoplasia and VATER association who required renal transplantation because of end‐stage renal failure. Additionally, congenital anomalies of the external iliac arteries are uncommon, with only five cases reported in a series of 8000 angiograms by Greebe 3. In our patient, the right external iliac artery was missing, and each branch of the internal iliac artery, including the internal pudendal artery branch of the abnormally running internal iliac artery, descended to the front of the pelvic floor. A replaced right hepatic artery (RRHA) generated from the superior mesenteric artery has been observed in 14.8% of the normal population 4. Therefore, it is difficult to determine whether this RRHA anomaly is specific to VATER association.

According to Tamisier et al.'s report 5, external iliac artery dysplasia is classified into three groups: (1) an anomaly of the running artery and origin of the external iliac artery, (2) obstruction or hypoplasia of the external iliac artery with persistent sciatic artery, and (3) external iliac artery aplasia or hypoplasia alone. In our patient, the external iliac artery anomaly was included in group 3. Although several reports of dysplasia of the iliac artery have been published, they were not related to VATER association.

Smith reported 6 that routine CT angiography found that 22.9% of recipients had a meaningful alteration in treatment based on angiographic findings before renal transportation. Same as renal transportation, CT angiography should be necessary before IVR treatment for the selection of cannulation route, especially who had a medical history of suspected VATER association. There is no information about the angle at which insertion becomes impossible because of kinking with the 4‐French sheath (RR‐A40G25A;Terumo), which we normally use. This time we selected the left femoral artery for cannulation. In emergency cases, it is not necessary to select the blood vessels that are considered difficult. However, if the cannulation site is confined to acute angular vessels, the Destination sheath (Terumo) should be chosen as it has high kink resistance from reinforcement by the coil.

## Conclusion

VATER syndrome is accompanied by many morphologic abnormalities that are unclear, and it is expected that many additional abnormalities of large running vessels due to VATER syndrome will be discovered. In such a case, careful selection of the cannulation route using pretreatment CT angiography is required.

## Authorship

TI: assisted in intravascular treatment. AT and MT: involved in the medical care of the patient. TI: supervised and corrected the text. All authors approved the final version of the manuscript.

## Informed Consent

The patient provided informed consent.

## Conflict of Interest

No conflict of interest.
